# The role of vitamin K and its antagonist in the process of ferroptosis-damaged RPE-mediated CNV

**DOI:** 10.1038/s41419-025-07497-0

**Published:** 2025-03-20

**Authors:** Xiaochan Dai, Xi Yang, Yifan Feng, Xinyuan Wu, Yahan Ju, Rong Zou, Fei Yuan

**Affiliations:** 1 Department of Ophthalmology, Zhongshan Hospital, Fudan University, Shanghai, 200032 PR China; 2 Department of Ophthalmology, Ninth People’s Hospital, Shanghai Jiao Tong University School of Medicine, Shanghai, 200011 PR China; 3 Shanghai Key Laboratory of Orbital Diseases and Ocular Oncology, Shanghai, 200011 PR China

**Keywords:** Mechanisms of disease, Diseases

## Abstract

Age-related macular degeneration (AMD) is the leading cause of irreversible vision loss in people over the age of 55. AMD currently affects approximately 8% of the world’s population, and the number is growing as the global population ages. Growing evidence suggests that pathological choroidal neovascularization (CNV) is often related to more severe and rapid vision loss and blindness associated with AMD. The typical clinical treatment is intravitreal injection of anti-vascular endothelial growth factor (anti-VEGF) agents. However, some patients do not respond well to this therapy, and the potential risks of long-term repeated injections cannot be ignored. Therefore, there is an urgent need to explore the specific mechanisms of CNV development and find new, safe, and effective treatments. In this study, our data indicate that ferroptotic damage of retinal pigment epithelium (RPE) and its induced VEGFA overexpression are critical promoting factors in the development of CNV. Vitamin K can mediate the protection of RPE cells from ferroptotic damage and regulate the expression of eIF2α-ATF4-VEGFA in a VKOR/FSP1-dependent manner, inhibiting new angiogenesis to alleviate CNV. On the contrary, vitamin K antagonists (VKA) represented by warfarin, can promote RPE ferroptotic damage and related vascular proliferation in mice and eventually aggravate CNV lesions. However, vitamin K still showed significant protective effects even in the presence of VKA. Due to its significant anti-ferroptosis and anti-neovascular effects, as well as its relative safety and convenience of use, vitamin K has excellent potential in the treatment of CNV and is expected to become a clinically effective and safe new CNV treatment strategy.

## Introduction

Age-related macular degeneration (AMD) stands as one of the leading causes of irreversible vision loss among the elderly population globally [1, 2]. With the aging of the population worldwide, the afflicted populace is expected to increase to 288 million by 2040 [3]. AMD manifests with chronic and progressive traits, categorizing into dry AMD, featuring geographic atrophy (GA), and wet AMD, characterized by choroidal neovascularization (CNV) in advanced stages. The latter is closely associated with heightened vision impairment and blindness [4, 5]. The main reason for the high blindness rate of wet AMD is that the abnormally high permeability of the characteristic pathological CNV leads to fundus bleeding, exudation, fibrous scarring, etc., profoundly impacting central vision. At present, vitreous injection of anti-vascular endothelial growth factor (anti-VEGF) agents stands as the standard treatment strategy in clinical practice. Nevertheless, some patients exhibit inadequate responses to this therapy, and the potential risks of prolonged repeated injections cannot be ignored. Considering the heightened risk of blindness and the limitations of therapy, it is urgent to find safe and effective treatments for pathological neovascularization.

Although the pathophysiology of CNV remains incompletely elucidated, it is widely accepted that the retinal pigment epithelium (RPE), serving as the interface between the neural retina and the choroid, plays a vital role in the development of CNV. RPE supports photoreceptor cells’ survival and physiological functions, maintaining homeostasis in the subretinal space by performing various physiological functions, including transport of fluids and substances, phagocytosis of photoreceptor outer segments, participation in blood-retinal barrier formation, and synthesis and secretion of various growth factors like vascular endothelial growth factor A (VEGFA) [6, 7]. Damage or dysfunction of RPE cells disrupts the homeostasis of pro-angiogenic factors and anti-angiogenic factors, potentially triggering a pathological angiogenic response, although the specific mechanism remains elusive.

Ferroptosis, first formally proposed in 2012 [8], denotes a new iron-dependent programmed cell death characterized by iron accumulation and lipid peroxidation [9]. Recent AMD-related studies showed that the accumulation of iron in the retina and RPE with age is a significant contributing factor to AMD development [10]. RPE are particularly susceptible to oxidative stress due to their location in the oxygen-rich retinal environment and their role in phagocytosing polyunsaturated fatty acid-rich outer segments of photoreceptors [11]. These results support the hypothesis that iron toxicity and oxidative stress-mediated ferroptosis-related RPE cell damage and death may significantly contribute to the occurrence and development of CNV, a key feature associated with severe vision loss in AMD. However, the relationship between ferroptosis-damaged RPE and CNV, along with the underlying mechanism remains unclear.

While various ferroptosis inhibitors have been discovered to effectively resist lipid peroxidation and cell death, none have yet received approval for clinical use. In recent years, some researchers have proposed vitamin K (VK) and its derivatives as cofactors of gamma-glutamyl carboxylase (GGCX), participating in coagulation-related physiological functions and exhibiting potent antioxidant properties. For example, VK can inhibit glutathione depletion-mediated oxidative cell death in neurons and oligodendrocytes and prevent oxidative cell death by preventing 12-lox activation and reactive oxygen species (ROS) generation [12].

In this study, we not only explored the relationship between RPE ferroptosis and the fate of CNV but also elaborated on the role of VK in protecting ferroptosis-damaged RPE, preserving its normal angiogenic function, and thereby alleviating CNV and its potential specific mechanisms. Besides enhancing the understanding of AMD’s pathophysiological mechanisms, our research also advocates for safer, more convenient, and effective therapeutic strategies to manage CNV and reduce severe vision loss.

## Results

### Abnormalities in RPE structure and function occur in laser-induced CNV in mice

**Fig. 1:**
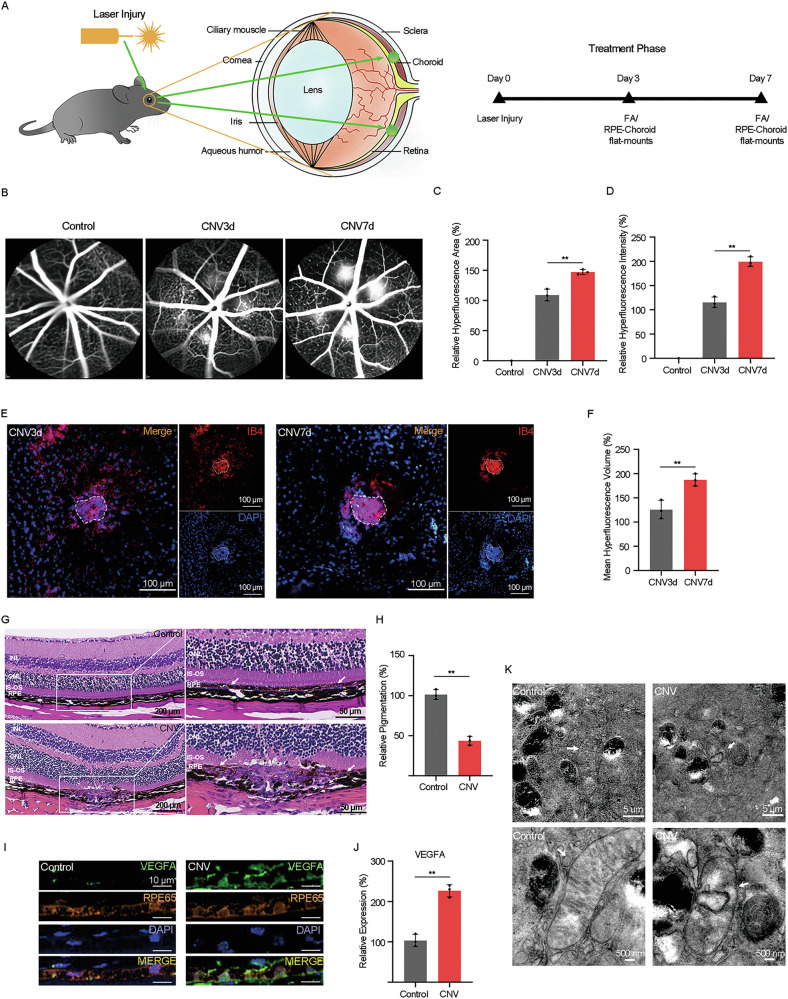
Abnormalities in RPE structure and function occur in laser-induced CNV in mice. **A** Schematic diagram of laser-induced CNV mice model. **B**–**D** The area and intensity of the hyperfluorescent lesions of CNV were measured by fluorescein angiography. **E**, **F** The volume of neovascularization of CNV were detected by Isolectin-B4 staining of RPE-choroidal flat mounts. **G**, **H** The morphological structure and pigment content of RPE in mice was detected by hematoxylin and eosin staining. **I**, **J** Protein level of VEGFA in RPE in mice treated as indicated were determined by immunofluorescence. **K** The Ultrastructure of mitochondria of RPE in mice treated as indicated was imaged by transmission electron microscopy. The data represent the averages of three independent experiments. Data shown are mean ± SD; Student’s *t* test; **P < 0.01. Scale bars: 100 μm (**E**); 200 μm (**G**); 10 μm (**I**); 5 μm, 500 nm (**K**).

To study the relationship between RPE damage and the occurrence and development of CNV in wet AMD, we initially established the widely used model of laser-induced CNV in mice (Fig. 1A). Subsequently, we performed fluorescein angiography (FA) and Isolectin-B4 (IB4, a biomarker of neovessels) staining of RPE-choroidal flat mounts to evaluate pathological vascular (Fig. 1B–F). Compared to day 3, the results on day 7 showed more apparent vascular leakage (Fig. 1B–D) and larger volume of neovascularization (Fig. 1E, F), indicating that the optimal observation model of CNV was established on day 7 post-laser photocoagulation.

Subsequently, we explored alterations in the morphological structure and physiological function of RPE in laser-induced CNV in mice. Hematoxylin and eosin staining (HE) revealed that the RPE layer in laser-injured lesions showed obvious depigmentation and abnormal morphological structure (Fig. 1G, H). In addition, immunofluorescence analysis revealed increased protein expression levels of VEGFA, a core regulator of neovascularization, in the RPE layer post-laser treatment (Fig. 1I, J). Interestingly, when observing the RPE by transmission electron microscopy (TEM), we found that RPE cells in CNV sample exhibited distinctive morphological features; specifically, mitochondria appeared smaller with heightened membrane density (mitochondria marked by white arrows, Fig. 1K), resembling typical mitochondrial features associated with ferroptosis.

### Ferroptosis-related damage occurs in RPE cells in laser-induced CNV in mice

**Fig. 2:**
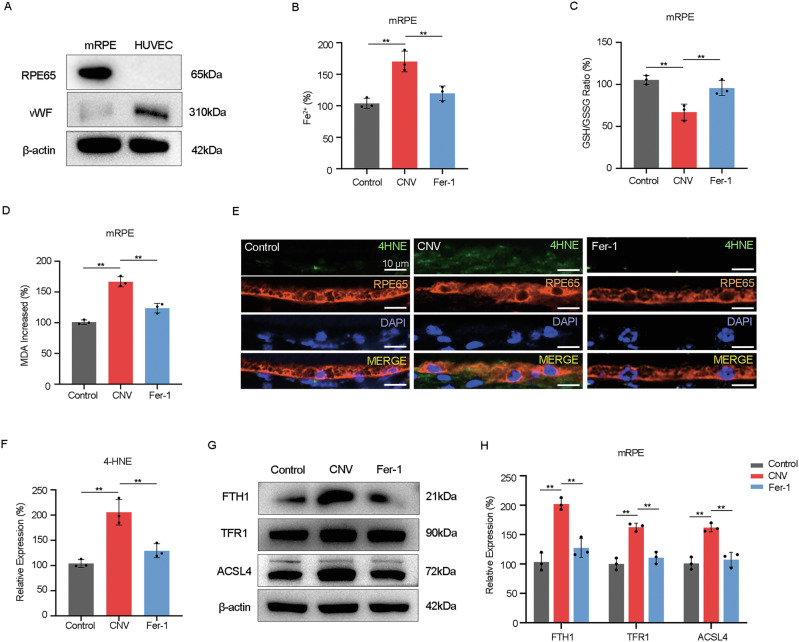
Ferroptosis-related damage occurs in RPE cells in laser-induced CNV in mice. **A** Protein levels of RPE65 (a marker of RPE) and vWF (a marker of endothelial cells) in mice-RPE and HUVEC were examined by western blot. **B**–**D** Level of Fe^2+^, ratio of GSH/GSSG, and level of MDA in RPE in mice treated as indicated were measured by detection kits. **E**, **F** Protein level of 4-HNE in RPE in mice treated as indicated was determined by immunofluorescence. **G**, **H** Protein levels of FTH1, TFR1, and ACSL4 in RPE in mice treated as indicated were examined by western blot. The data represent the averages of three independent experiments. Data shown are mean ± SD; one-way ANOVA with Bonferroni correction; **P < 0.01. Scale bars: 10 μm.

Ferroptosis is a kind of iron-dependent programmed cell death characterized by iron overload and accumulation of lipid peroxides [9]. To ascertain the association between the damage of RPE in CNV and ferroptosis, mice were subjected to ferroptosis inhibitor ferrostatin-1 (Fer-1). Mouse RPE cells were isolated and identified for subsequent experiments (Fig. 2A).

The results showed that Fer-1 alleviated the accumulation of excessive Fe^2+^, a hallmark of ferroptosis, in RPE of CNV mice (Fig. 2B). Meanwhile, Fig. 2C–F showed that the decreased ratio of GSH (a crucial intracellular antioxidant)/GSSG and the increased levels of lipid peroxidation markers, 4-hydroxynonenal (4-HNE), and malondialdehyde (MDA), a final product of polyunsaturated fatty acid peroxidation, in the RPE layer of CNV mice were significantly reversed under Fer-1 treatment.

Subsequently, we further measured the expression levels of several ferroptosis-related proteins, including FTH1, TFR1, and ACSL4, in RPE of CNV mice. FTH1, a critical subunit of ferritin, facilitates the oxidation of harmful ferrous iron to maintain cellular homeostasis [13]; TFR1 facilitates cellular uptake of transferrin-bound iron [14]; and ACSL4 aids in integrating polyunsaturated fatty acids into phospholipids, promoting lipid peroxidation [15]. Our results showed upregulated expression levels of these proteins in RPE of CNV mice (Fig. 2G, H), suggesting abnormal intracellular iron homeostasis, heightened cellular uptake of transferrin-bound iron, and enhanced intracellular lipid peroxidation levels. As anticipated, the expression levels of these three ferroptosis-related proteins were significantly reversed in the Fer-1 treatment group.

### VK protects RPE from ferroptosis in vitro

Recently researchers have discovered VK’s effectiveness in inhibiting lipid peroxidation [12], a typical feature of ferroptosis. To explore whether VK offers potential protective effect against ferroptotic damage in RPE in vitro, we used the typical ferroptosis inducer, erastin, to induce ferroptosis injury in ARPE-19 cells, followed by assessment of changes in cell viability, morphological structure, and ferroptosis-related marker levels.

**Fig. 3:**
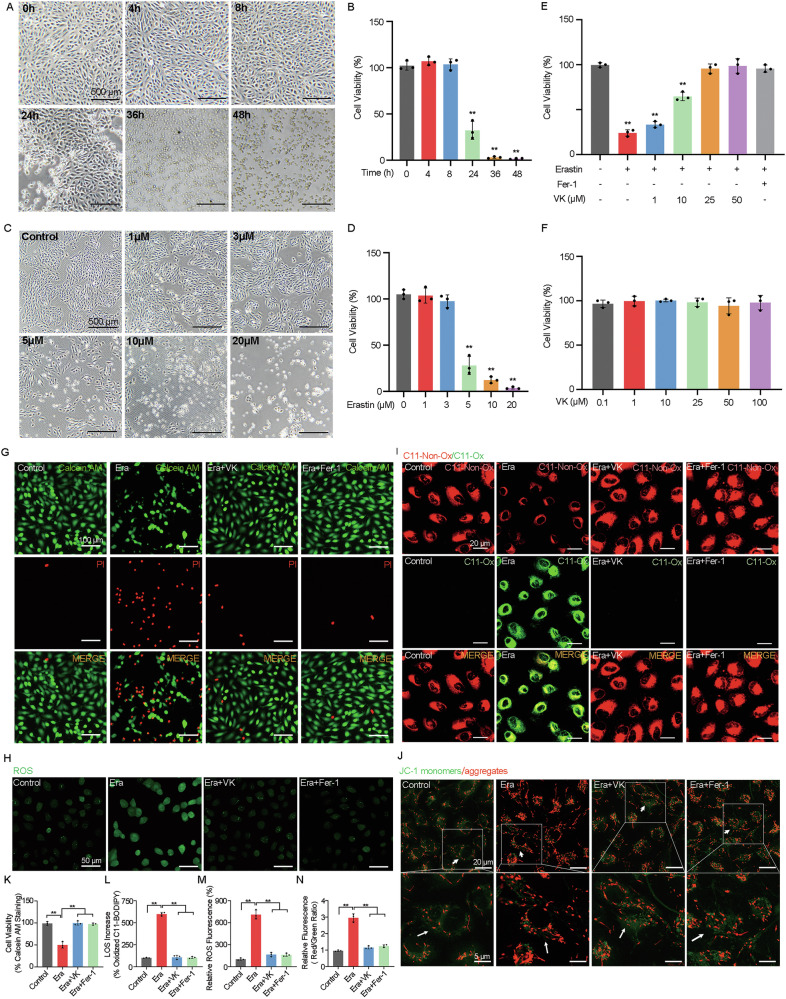
Vitamin K protects RPE from ferroptosis in vitro. **A**, **C** Morphological changes of ARPE-19 cells were observed by optical microscopy. **B**, **D** Cell viability of ARPE-19 cells was detected by CCK-8 assays. **A**, **B** ARPE-19 cells were treated with erastin (5 μM) for 0, 4, 8, 24, 36 and 48 h, respectively. **C**, **D** ARPE-19 cells were treated with erastin at concentrations of 0, 1, 3, 5, 10, 20 μM, respectively, for 24 h. **E**, **F** Cell viability of ARPE-19 cells was detected by CCK-8 assays. **E** ARPE-19 cells were treated with erastin (5 μM) for 24 h after pretreatment with/without VK (1, 5, 10, 25 μM) or Fer-1 (10 μM) for 1 h. **F** ARPE-19 cells were treated with VK at concentrations of 0.1,1, 10, 25, 50, 100 μM respectively for 24 h. **G**–**I** ARPE-19 cells were treated with erastin (5 μM) for 24 h or **J** 4 h after pretreatment with/without VK (10 μM) or Fer-1 (10 μM). **G**, **K** Cell survival/death and cell mortality qualification of ARPE-19 cells were detected by live/dead staining (Calcein AM: Live cells; PI: Dead cells). **H**, **L** ROS and **I**, **M** lipid ROS production in ARPE-19 cells were detected using a DCFH-DA-ROS assay kit and BDP 581/591 C11 fluorescent probe (Green: oxidized BDP 581/591 C11; Red: non-oxidized BDP 581/591 C11) respectively. **J**, **N** Changes in mitochondrial membrane potential in ARPE-19 cells were evaluated by mitochondrial membrane potential probe JC-1 (Red: high potential; Green: low potential). The data represent the averages of three independent experiments. Data shown are mean ± SD; one-way ANOVA with Bonferroni correction; **P < 0.01. Scale bars: 500 μm (**A**, **C**); 100 μm (**G**); 50 μm (**H**); 20 μm (**I**, **J**); 5 μm (**J**).

Given that we found significant alterations in cell viability and morphological structure of ARPE-19 cells (Fig. 3A–D), we use 5 μM-24 h-erastin incubation to induce ferroptotic damage in ARPE-19 cells.

To investigate the rescue effects of VK1, cell viability was determined via CCK-8 analysis and live/death staining. VK1 pretreatment demonstrated a significant dose-dependent rescue effect on erastin-induced cell death, eventually achieving a protective effect comparable to Fer-1 (Fig. 3E). Satisfactorily, incubation with VK1 at doses up to 100 μM for 24 h exhibited no significant impact on the cell viability of ARPE-19 cells (Fig. 3F). The results of the following live/death staining were consistent with that of CCK-8 assay (Fig. 3G, K).

Subsequently, we evaluated the effects of VK1 on intracellular levels of lipid peroxidation levels in erastin-treated ARPE-19 cells. ROS (Fig. 3H, L) and BDP 581/591 C11 fluorescent staining (Fig. 3I, M) showed pretreatment with VK1 can effectively improve erastin-induced elevation in lipid peroxidation levels, a response like Fer-1 pretreatment.

Notably, we also used the mitochondrial membrane potential probe JC-1 (Fig. 3J, N) to assess mitochondrial function in ARPE-19 cells. We found that both VK1 and Fer-1 can inhibit the mitochondrial hyperpolarization process induced by erastin in ARPE-19 cells.

### VK regulate the angiogenic function of ferroptosis-damaged RPE

**Fig. 4:**
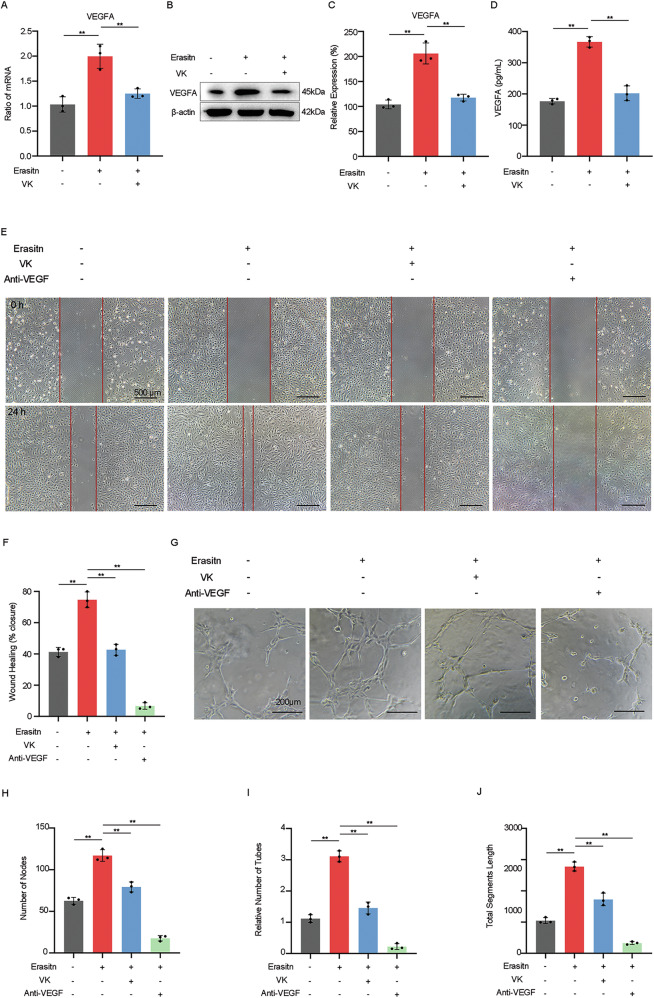
Vitamin K regulate the angiogenic function of ferroptosis-damaged RPE. **A** mRNA and **B**, **C** protein expression levels of VEGFA in erastin-treated ARPE-19 cells with/without pretreatment of VK were determined by qPCR and western blot respectively. **D** The secretion levels of VEGFA from erastin-treated ARPE-19 cells with/without pretreatment of VK were determined by quantitative ELISA. HUVEC were treated with RPE conditioned culture medium obtained from untreated ARPE-19 (NC-CM), erastin-treated ARPE-19 cells with/without VK pretreatment (Era-CM, Era+VK-CM), and Era-CM with Anti-VEGFA (0.3125 mg/mL, Era+Anti-VEGFA-CM). **E**, **F** Migration capacity of HUVEC was detected by scratch migration assay. **G**–**J** Tube formation ability of HUVEC was detected by tube formation assay. The numbers of nodes, relative number of tubes, and total segments length were quantified by ImageJ. The data represent the averages of three independent experiments. Data shown are mean ± SD; one-way ANOVA with Bonferroni correction; **P < 0.01. Scale bars: 500 μm (**E**); 200 μm (**G**).

Since RPE synthesis and secretion of angiogenesis-related factors play a vital role in the progression of CNV, we evaluated the impact of VK on regulating angiogenesis function in ferroptotic-damaged RPE. RT-qPCR (Fig. 4A), western blot (Fig. 4B, C) and ELISA (Fig. 4D) results showed that following erastin stimulation, the gene and protein expression level of VEGFA, a core regulator of neovascularization, significantly increased in ARPE-19 cells but restored under VK1 pretreatment.

To further investigate the effects of VK on the angiogenic behavior of endothelial cells mediated by ferroptosis-damaged RPE, we used conditioned culture medium (CM) obtained from ARPE-19 to culture human umbilical vein endothelial cells (HUVEC), and then evaluated the migration and tube formation abilities of HUVEC through scratch and tube formation assays. The results revealed that compared to Era-CM, the promoting effects on HUVEC migration (Fig. 4E, F) and tubule formation function (Fig. 4G–J) were significantly weakened in the Era+VK-CM group and Era+Anti-VEGFA-CM group.

### The anti-angiogenic effects of VK on ferroptosis-damaged RPE are closely related to ATF4

**Fig. 5:**
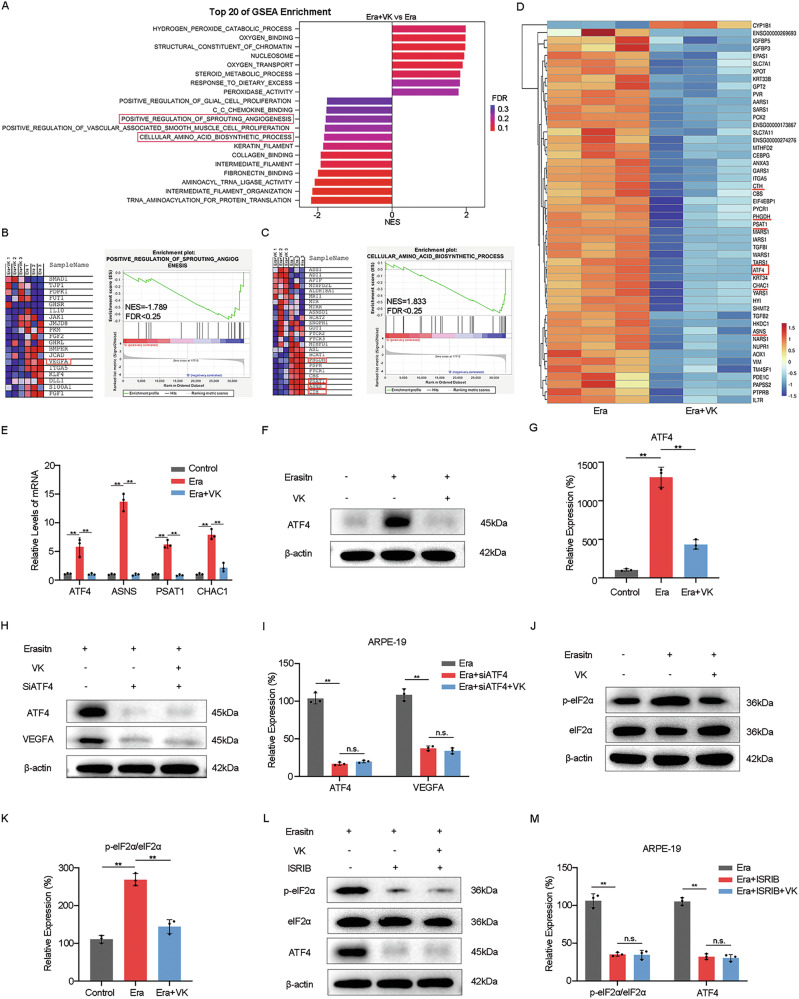
The anti-angiogenic effects of Vitamin K on ferroptosis-damaged RPE are closely related to ATF4. **A**–**D** RNA-sequencing analysis of erastin-treated ARPE-19 cells. Cells were pretreated with/without VK (10 μM) before treated with erastin (5 μM) for 24 h. **A** The top 20 enriched altered gene sets between the Era and Era+VK groups analyzed by Gene Set Enrichment Analysis (GSEA). **B**, **C** GSEA analysis between Era and Era+VK groups of the “ positive regulation of sprouting angiogenesis “ gene set and “ cellular amino acid biosynthetic process “ gene set in the GO database. The heat map shows the top enriched genes. **D** The heat map showed RNA-seq expression of the top 50 marker genes identified from Era and Era+VK groups. **E** mRNA levels of ATF4, ASNS, PSAT1 and CHAC1 in the ARPE-19 cells were determined by qPCR. **F**, **G** Protein level of ATF4 in erastin-treated ARPE-19 cells with/without pretreatment of VK (10 μM) were measured by western blot. **H**, **I** Protein levels of ATF4 and VEGFA in erastin-treated ARPE-19 cells with/without transfection of siATF4 or pretreatment of VK (10 μM) were measured by western blot. **J**, **K** Protein levels of p-eIF2α and eIF2α in erastin-treated ARPE-19 cells with/without pretreatment of VK (10 μM) were measured by western blot. **L**, **M** Protein levels of p-eIF2α, eIF2α, and ATF4 in erastin-treated ARPE-19 cells with/without pretreatment of VK (10 μM) or ISRIB (10 μM) were measured by western blot. The data represent the averages of three independent experiments. Data shown are mean ± SD; one-way ANOVA with Bonferroni correction; **P < 0.01, n.s. not significant.

Having observed that VK can inhibit the abnormal proangiogenic response of ferroptosis-damaged RPE, we proceeded to conduct transcriptome sequencing (RNA-seq) analysis on erastin-treated ARPE-19 cells with/without VK1 pretreatment to further explore the underlying mechanism of this regulation. Through Gene set enrichment analysis (GSEA), we found that VK1 significantly influenced genes related to positive regulation of sprouting angiogenesis and cellular amino acid biosynthetic processes in ferroptosis-damaged RPE cells (Fig. 5A). Further study revealed a significant downregulation of VEGFA (Fig. 5B) and several downstream signature genes of ATF4 (Fig. 5C) following VK1 treatment. It is gratifying that in subsequent differential gene analysis, we found that ATF4 related target genes (ASNS, PSAT1, CHAC1, etc.) decreased in the VK1 pretreatment group (Fig. 5D), which were verified with subsequent RT-qPCR (Fig. 5E). Western blot results (Fig. 5F, G) also confirmed that VK1 has a significant effect on alleviating the increase of ATF4 protein levels in erastin-treated ARPE-19 cells, suggesting that ATF4 is likely to play an essential role in VK1 regulating the angiogenic function of ferroptosis-damaged ARPE-19 cells.

To validate this hypothesis, we knocked down ATF4 by transfecting siATF4 (Supplementary Fig. S1A). As shown in Fig. 5H, I, the protein expression level of VEGFA in erastin-treated ARPE-19 cells decreased upon the transfection of siATF4, with no significant difference between pretreated with/without VK1.

ATF4 is recognized as a critical transcription factor to various stresses, which are typically mediated by the phosphorylation of eukaryotic initiation factor 2 (eIF2α) [16]. We then examined the phosphorylation status of eIF2α (p-eIF2α) in erastin-treated ARPE-19 cells. Results showed that erastin stimulation increased the protein level p-eIF2α in cells, which were significantly inhibited by VK1 pretreatment (Fig. 5J, K), suggesting that VK1 regulates protein expression level of ATF4 in ferroptosis-damaged ARPE-19 cells possibly by regulating the phosphorylation of eIF2α.

To further verify this hypothesis, we used ISRIB to reverse the effects of eIF2α phosphorylation. The results showed that the high protein levels of p-eIF2α and ATF4 induced by erastin were significantly blocked by ISRIB. No significant difference was found in the protein expression level of ATF4 in erastin-treated ARPE-19 cells regardless of whether they were pretreated with VK1 (Fig. 5L, M).

### Effects of VK antagonists (VKA) on VK resistance to the ferroptosis damage in RPE

Although our results demonstrate the potential of VK in the treatment of CNV, the fact that VKA, represented by warfarin, have been widely used in clinical therapy of thromboembolic diseases cannot be ignored [17–21]. Similar to AMD, many thromboembolic-related diseases are age-correlated, raising the likelihood of concurrent VKA use among AMD patients.

**Fig. 6:**
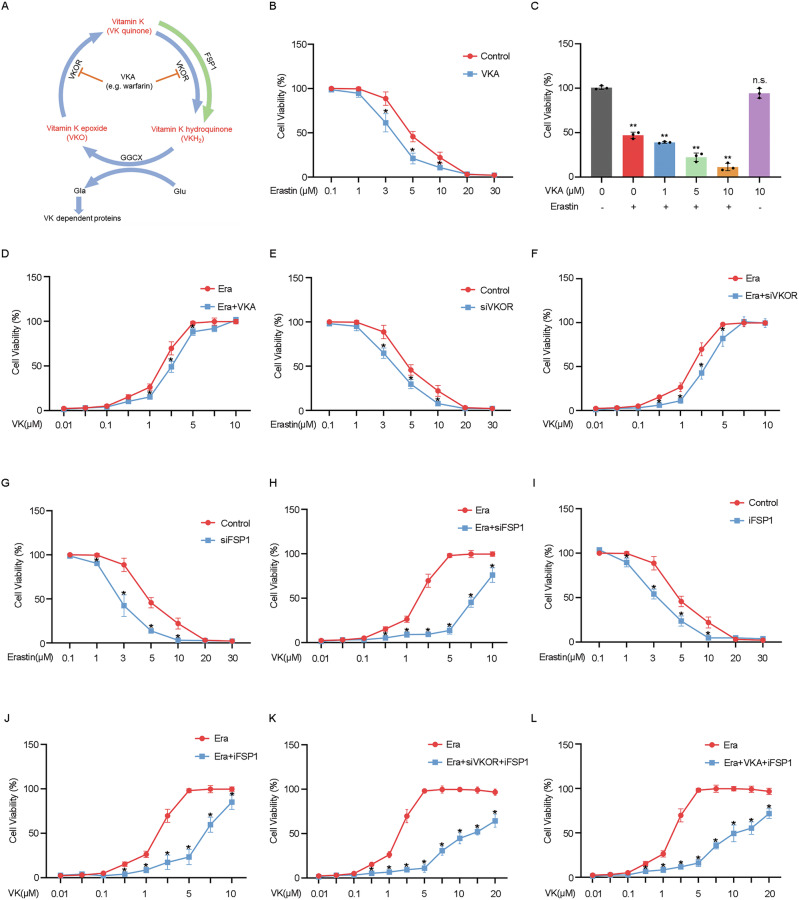
Effects of vitamin K antagonists on vitamin K resistance to the ferroptosis damage in RPE. **A** Schematic diagram of the vitamin K cycle. **B**–**L** Cell viability of ARPE-19 cells was detected by CCK-8 assays. ARPE-19 cells were treated with erastin (5 μM) for 24 h with/without **B**–**D** pretreatment of warfarin (10 μM, VKA) or VK (10 μM); **E**, **F** transfection of siVKOR or pretreatment of VK (10 μM); **G**, **H** transfection of siFSP1 or pretreatment of VK (10 μM); **I**, **J** pretreatment of iFSP1 (10 μM) or VK (10 μM); **K** transfection of siVKOR and pretreatment with iFSP1 (10 μM) and VK (10 μM); **L** pretreatment with VKA (10 μM), iFSP1 (10 μM), and VK (10 μM), as indicated. The data represent the averages of three independent experiments. Data shown are mean ± SD; one-way ANOVA with Bonferroni correction; *P < 0.05, **P < 0.01, n.s. not significant.

VK hydroquinone (VKH_2_), derived from VK through the classical circulation pathway, acts as a cofactor of GGCX, catalyzing carboxylation of VK-dependent proteins including coagulation factors, thereby exerting a coagulation effect [22]. VK epoxide reductase (VKOR), not only catalyzes the conversion of VK into active VKH_2_ but also converts VK epoxide (VKO) formed post VKH_2_ oxidation in GGCX-mediated reaction back into VK [23], ensuring the recycling of VK (Fig. 6A). VKA block the VK cycle by inhibiting VKOR, thereby inhibiting the activation of coagulation factors to exert an anticoagulant effect [17].

To explore the effects of VKA, represented by warfarin, on ferroptosis-damaged RPE, we pretreated ARPE-19 cells with warfarin prior to erastin treatment.CCK-8 results showed that warfarin pretreatment exacerbated ARPE-19 cell death in a concentration-positive correlation (Fig. 6B, C) and weakened the sensitivity of ferroptosis-damaged RPE to VK1 (Fig. 6D), suggesting that VK’s anti-ferroptosis effect may rely on VKOR. However, even in the presence of warfarin, high VK1 doses could ultimately rescue ARPE-19 from cell death (Fig. 6D), hinting at VK’s concurrent use with warfarin.

To validate these findings and explore the specific mechanism, we knocked down VKOR by siRNA. Since there are two forms of VKOR in vertebrates, VKORC1 and VKORC1L1 [23], we simultaneously knocked them down (Supplementary Fig. S1B). CCK-8 assay results showed that VKOR knockdown exacerbated ARPE-19 cell death (Fig. 6E) and attenuated the sensitivity of ferroptosis-damaged RPE to VK1 (Fig. 6F), verifying VKOR’s integral role in VK’s resistance to RPE ferroptotic damage. Similar to warfarin treatment, high VK1 doses could fully rescue ARPE-19 from erastin-induced cell death (Fig. 6F), suggesting VK may also mitigate ferroptosis-damage through other pathways.

Recent literature suggests FSP1 can reduce VK to form VKH_2_ when VKOR is inhibited, allowing normal physiological VK functions [24]. To investigate the role of FSP1 in VK’s inhibition of RPE ferroptotic damage, we knocked down FSP1 by transfecting siRNA (Supplementary Fig. S1C), and used iFSP1 to inhibit FSP1 pharmacologically. The results showed that both siFSP1 and iFSP1 can exacerbate ARPE-19 cell ferroptosis and weaken the ability of VK1 to inhibit ARPE-19 cell ferroptosis (Fig. 6G–J), confirming FSP1’s involvement in VK anti-RPE ferroptosis injury process.

Furthermore, inhibiting both VKOR and FSP1 significantly hampered VK1’s protective effect on ferroptosis-damaged ARPE-19, even at a concentration of VK1 as high as 20 μM (Fig. 6K). Simultaneously, similar experimental results were obtained when pretreated with warfarin and iFSP1 (Fig. 6L), underscoring VKOR/FSP1’s crucial role in VK’s protection against RPE ferroptotic damage.

### Effects of VKA on VK mediated anti-angiogenic function of ferroptosis-damaged RPE

**Fig. 7:**
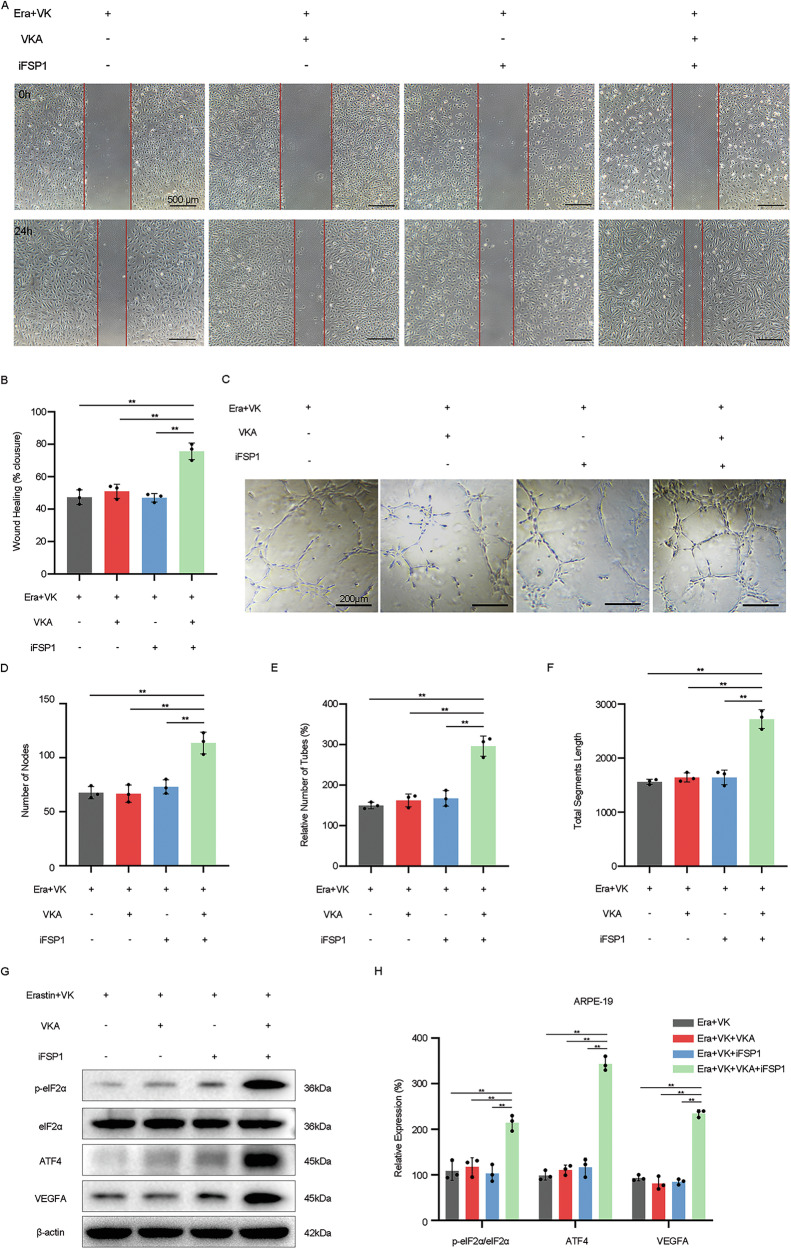
Effects of Vitamin K Antagonists on Vitamin K mediated anti-angiogenic function of ferroptosis-damaged RPE. **A**–**F** HUVEC were treated with RPE conditioned culture medium collected from erastin-treated ARPE-19 cells with/without VK (10 μM), VKA (10 μM), and iFSP1 (10 μM) pretreatment (Era+VK-CM, Era+VK + VKA-CM, Era+VK+iFSP1-CM, Era+VK+Warf+iFSP1-CM). **A**, **B** Migration capacity of HUVEC was detected by scratch migration assay. **C**–**F** Tube formation ability was detected by tube formation assay. The numbers of nodes, relative number of tubes, and total segments length were quantified by ImageJ. **G**, **H** Protein levels of p-eIF2α, eIF2α, ATF4, and VEGFA in erastin-treated ARPE-19 cells pretreated with/without VK (10 μM), VKA (10 μM), and iFSP1 (10 μM) were measured by western blot. The data represent the averages of three independent experiments. Data shown are mean ± SD; one-way ANOVA with Bonferroni correction; **P < 0.01, n.s. not significant. Scale bars: 500 μm (**A**); 200 μm (**C**).

To further explore the effects of VKOR/FSP1 on VK’s regulation of the angiogenic function of ferroptosis-damaged RPE, we used CM collected from erastin-treated ARPE-19 cells with/without warfarin, iFSP1, and VK1 pretreatment to culture HUVEC. The results showed that compared to Era+VK-CM, HUVEC cultured with Era+VK + VKA+iFSP1-CM exhibited enhanced migration (Fig.7A, B) and tubular formation ability (Fig. 7C–F). In contrast, HUVEC in either Era+VK + VKA-CM or Era+VK+iFSP1-CM group have similar abilities of migration and tube formation to those in the Era+VK-CM group.

Subsequently, we detected changes in pathways related to the angiogenesis function of ARPE-19 cells through western blot. Figure 7G, H showed that warfarin+iFSP1 rendered VK1 ineffective in reducing the protein levels of p-eIF2α, ATF4, and VEGFA in ferroptosis-damaged ARPE-19 cells, while warfarin or iFSP1 alone had no significant effect on this, suggesting that VKOR/FSP1 also plays an essential role in VK regulating the angiogenic function of ferroptosis-damaged RPE.

### VK effectively alleviates the progression of laser-induced CNV in mice

**Fig. 8:**
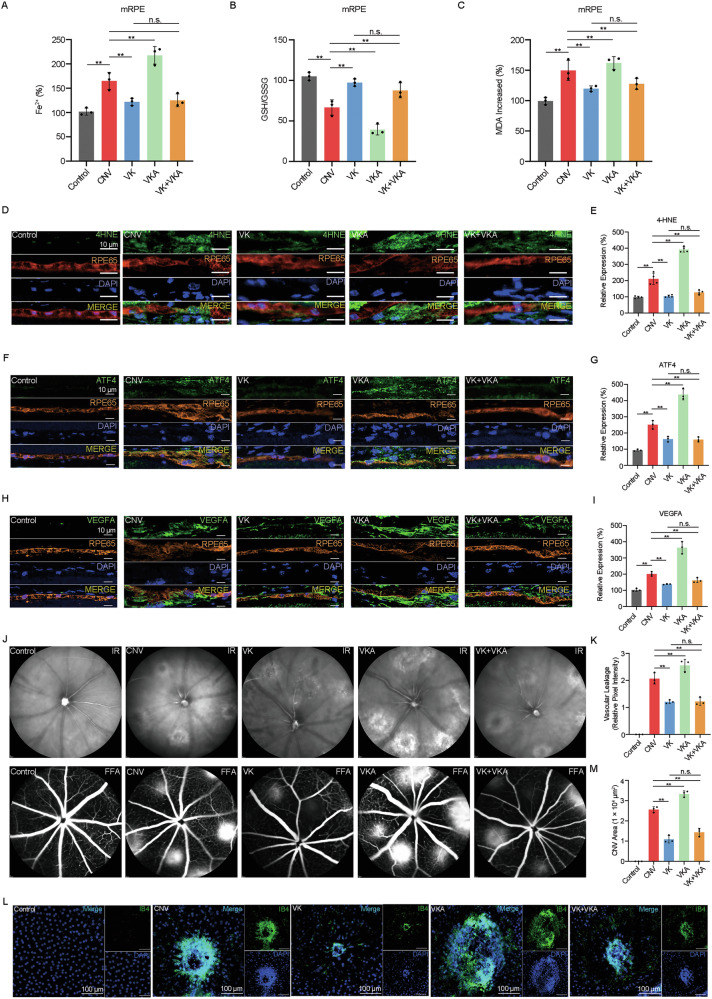
Vitamin K effectively alleviates the progression of laser-induced CNV in mice. Mice were treated with/without VK and VKA at determined doses and time points after laser irradiation. **A**–**C** Level of Fe^2+^, ratio of GSH/GSSG, and level of MDA in RPE in mice were measured by detection kits. **D**–**I** Protein levels of 4-HNE, ATF4, and VEGFA in RPE in mice were determined by immunofluorescence. **J**, **K** The area and intensity of the hyperfluorescent lesions of CNV were measured by fluorescein angiography. **L**, **M** The volume of neovascularization of CNV were detected by Isolectin-B4 staining of RPE-choroidal flat mounts. The data represent the averages of three independent experiments. Data shown are mean ± SD; one-way ANOVA with Bonferroni correction; **P < 0.01, n.s. not significant. Scale bars: 10 μm (**D**, **F**, **H**); 100 μm (**L**).

Building upon our results of in vitro studies, we further explored the role of VK1 and warfarin in the development of CNV in vivo. Our results show that VK1 significantly restored the abnormal level of Fe^2+^, GSH, MDA, and 4-HNE (Fig. 8A–E), and reduced the expression levels of ATF4 and VEGFA proteins in RPE of CNV lesions (Fig. 8F–I). Subsequently, FA (Fig. 8J, K) and RPE-choroidal flat mounts (Fig. 8L, M) results showed that the CNV area and vascular leakage were reduced after VK1 treatment. However, warfarin exhibited the exact opposite effects to VK1 (Fig. 8A–M).

It is noteworthy that when treated with VK1 and warfarin together, both the degree of ferroptotic damage (Fig. 8A–E) and the expression levels of angiogenic factors (Fig. 8F–I) in RPE cells were similar to those treated with VK1 alone, and the CNV area and vascular leakage (Fig. 8J–M) still exhibited a decreasing trend.

## Discussion

Although the pathogenesis of CNV remains unclear, researchers believed that the injury of RPE, an essential source of VEGFA, can disrupt the balance of pro- and anti-angiogenic factors and aggravating of pathological neovascularization [25, 26]. However, the specific damage characteristics of RPE in CNV have not been fully elucidated. In this study, we found that the RPE cells in CNV lesions exhibited abnormal mitochondrial structure, accompanied by increased VEGFA production. Notably, RPE cells also showed significant depigmentation, which was proposed to result in the release of reactive iron leading to lipid peroxidation and cytotoxicity [27–29]. This was confirmed by the subsequent accumulation of iron and lipid peroxides, and increased levels of ferroptosis-related protein were also found, which were ameliorated by Fer-1. Subsequent in vitro studies revealed VEGFA expression upregulated in ferroptosis-damaged RPE cells, which could promote endothelial cell tube formation. Based on these results, the development of CNV may be closely related to ferroptosis-damage RPE.

In recent years, the antioxidant effect of VK has been discovered, some scholars have proposed VK as an effective agent against lipid peroxidation [10, 12, 30]. Natural forms of VK include VK1 and VK2. Considering VK1 is currently the only VK preparation approved by the National Medical Products Administration (NMPA), which is widely used in clinical practice, we focused on VK1 as the research object. Experimental results confirmed that VK exhibits nearly equivalent efficacy as the classic ferroptosis inhibitor Fer-1 in mitigating RPE ferroptosis damage. It maintains RPE cell viability and standard morphological structure while preventing increases in ferrous iron and lipid oxidation levels. Meanwhile, we found that the level of VEGFA increased excessively in ferroptosis-damaged RPE cells and caused hyperstimulation of the co-cultured endothelial cells, which could be effectively alleviated by VK supplementation. This suggests that VK can not only alleviate the symptoms of ferroptosis but also alleviate hyperangiogenesis, demonstrating anti-vascular function. Encouragingly, VK also shows good safety profiles, with no cytotoxicity observed even at a high dose of 100 μM, meeting the primary requirement for clinical application of drugs.

ATF4, a basic leucine zipping (bZIP) transcription factor, is widely expressed in all organs of the human body and responds to various stress signals such as hypoxia, endoplasmic reticulum stress, amino acid deprivation, and oxidative stress through the mediation of eIF2α, a core factor in stress response [31]. In recent years, with the deepening of research, the role of ATF4 related to angiogenesis has also become apparent. Some researchers found that ATF4 is related to VEGFA expression in the retina [32, 33]. In addition, another VK-related study found that VK supplements had a regulatory effect on ATF4 protein levels in the arteries of high-salt mice [34], suggesting a close relationship between VK, eIF2α-ATF4 and VEGFA. In this study, we determined the causal relationship between eIF2α-ATF4 and VEGFA in ferroptosis-damaged RPE cells and the regulatory role of VK. The results showed that ferroptosis-damaged RPE could up-regulate the protein expression of VEGFA by activating the eIF2α-ATF4 pathway, while VK pretreatment could inhibit the overexpression of eIF2α-ATF4-VEGFA, thereby inhibiting neovascularization. Consistently, we subsequently found that the overexpression of ATF4 and VEGFA in RPE of CNV lesions could be inhibited by VK. Our study shows that VK can maintain the stability of VEGFA by inhibiting the activation of the eIF2α-ATF4 pathway in ferroptosis-damaged RPE, thereby inhibiting the development of CNV, highlighting the potential of VK as a new clinical treatment strategy for CNV.

It has been understood that the reduction of VK to VKH_2_ via VKOR is essential for the normal physiological function of VK, and VKOR is the target of VKA. Given that many AMD patients require long-term anticoagulant therapy with VKA, such as warfarin, we need to be vigilant about the potential problems VKA may pose when using VK to prevent CNV. In this study, after observing that VK resists RPE ferroptosis and related hyperangiogenesis in vitro and inhibits CNV in vivo, our experiments further found that warfarin exacerbates RPE ferroptosis damage and the development of CNV. Nevertheless, the detrimental effects of warfarin could be eliminated when VK was sufficient. Even in the presence of warfarin, VK still protect mice from CNV effectively.

The underlying mechanism behind this phenomenon may be related to FSP1. FSP1 is an NAD(P)H-dependent ubiquinone oxidoreductase. It was recently discovered that FSP1 can consume NADPH to exert VK reductase activity when VKOR is inhibited. When VK is sufficient, VK is reduced to VKH_2_ by FSP1 to exert normal physiological functions. Our results show that inhibiting FSP1 or VKOR alone does not compromise the effect of VK in ferroptosis-damaged RPE. However, when iFSP1 and warfarin were added simultaneously, VK’s ability of anti-ferroptosis, regulating eIF2α-ATF4-VEGFA pathway, and anti-CNV was significantly diminished. This further confirmed the vital role of VKOR/FSP1 in mediating VK’s anti-ferroptosis, anti-angiogenesis, and protection of CNV.

In summary, this study reveals that RPE ferroptotic damage and the resultant VEGFA overexpression are key factors in promoting the occurrence and development of CNV. VK can protect RPE cells from ferroptosis damage and regulate the expression of eIF2α-ATF4-VEGFA in a VKOR/FSP1-dependent manner, thereby inhibiting new angiogenesis to alleviate CNV (Fig. S2). On the contrary, warfarin not only synergistically promotes ferroptotic damage of RPE and subsequent hyperangiogenesis but also exacerbates CNV lesions in mice. However, despite this, VK still demonstrated significant protective effects in the presence of warfarin. This evidence indicates that VK holds excellent potential for treating CNV due to its significant anti-ferroptosis and anti-neovascular effects, as well as its relatively safe and convenient use.

However, our experiment still has limitations. Although ARPE-19 cells have long been widely used to study the physiological functions and pathological mechanisms of RPE, there is still a gap between them and fetal human RPE cells or native RPE cells. For example, ARPE-19 cells have reduced ability to express certain key proteins, weak tight junction function, lack of typical hexagonal shape and extensive pigmentation, and loss of certain RPE-specific metabolic functions (such as lack of acyl-transferase, -hydrolase, and 11-cis isomerase activities) [35–37]. In particular, ARPE-19 cells have less VEGFA secretion, and their apical secretion state also differs from the characteristics of basal secretion of fetal human RPE cells or native RPE cells [36]. In this study, we used in vitro experiments to verify the in vivo results of ARPE-19 cells, However, fetal human RPE cells may be more worthy of our attempts in future studies to further validate our findings.

## Materials and methods

### Reagents

Erastin (a ferroptosis activator), Ferrostatin-1(Fer-1, a ferroptosis inhibitor), Anti-VEGFA (Ranibizumab), iFSP1 (a selective and glutathione-independent inhibitor of ferroptosis suppressor protein 1), and ISRIB (a reagent that can effectively reverse the phosphorylation of eukaryotic initiation factor 2α) were obtained from Selleck Chemicals (Houston, TX, USA). Vitamin K1, warfarin, and warfarin sodium were purchased from Aladdin Scientific (Shanghai, China). DMEM medium, fetal bovine serum (FBS), penicillin/streptomycin, bovine serum albumin (PBS), Hanks’ Balanced Salt Solution (HBSS), and Live/dead Kit were purchased from Invitrogen (Carlsbad, CA, USA). Paraformaldehyde (PFA), bovine serum albumin (BSA), and Triton X-100 were obtained from Beyotime (Jiangsu, China). The Iron Assay Kit, GSH/GSSG Quantification Kit, Cell Counting Kit-8 (CCK-8) reagent, Malondialdehyde (MDA) Assay Kit, Lipid Peroxidation Probe BDP 581/591 C11, Highly Sensitive DCFH-DA-ROS Assay Kit, and JC-1 MitoMP Detection Kit were purchased from Dojindo (Kumamoto, Japan).

### Ethics statement

All the rearing and experimental procedures adhered to the Association for Research in Vision and Ophthalmology Statement and the ARRIVE guidelines and were approved by the Ethical Committee on Animal Experiments of Animal Care Committee of Zhongshan Hospital, Fudan University (No. 2019-285), Shanghai, China.

### Animals

Male C57BL/6J mice (6–8 weeks old; 20 ~ 25 g) were purchased from SLAC Laboratory Animal (Shanghai, China). The animals underwent a 1-week acclimation period prior to any experimental procedures. All animals were housed in standard mouse cages (370 × 270 × 170 mm) with 3 mice per cage, provided with standard food and clean water ad libitum. All mice were monitored twice daily for survival throughout the experiment.

### Laser-induced CNV model establishment and treatment

CNV was induced by photocoagulation, following established protocols [38]. Briefly, mice were anesthetized with 2% sodium pentobarbital (30 mg/kg; Sigma-Aldrich, St. Louis, MO, USA), and anesthetic depth was evaluated by the loss of the righting reflex. Pupil dilation was achieved using 1% tropicamide (Alcon Laboratories, Ft. Worth, TX, USA). Laser photocoagulation (532 nm, 120 mW, 50-μm spot size, 100 ms) was performed bilaterally using a diode-pumped solid-state laser (VISULAS 532 s; Carl Zeiss Meditec, Jena, Germany). 20 laser spots per eye were applied around the optic nerve for the western blot, while 4-6 laser spots per eye were applied for other experiments. Following laser irradiation, mice were placed on a heating plate at 37 °C until they regained consciousness. After a predetermined time, mice were euthanized for subsequent experiments by intraperitoneal injection of an overdose of 2% sodium pentobarbital (200 mg/kg) following spectral domain optical coherence tomography and fundus fluorescein angiography. The eyes were enucleated, and the neuroretina underlying the RPE were carefully separated to form eye cups.

Mouse RPE cells were separated from other structures through enzymic digestion (hyaluronidase and dispase; Medchem Express, Monmouth Junction, NJ, USA) as previous described [6]. Specifically, eye cups were digested with enzymic at 37 °C for 30 min, then washed with BSA/PBS, followed by mechanical dissection. The RPE cells identification was performed by Western blot (Fig. 2A).

### In vivo research design and drug management

In the first in vivo experiment, C57BL/6J mice were randomly assigned to three groups: (1) Control group (*n* = 6), (2) CNV3d group (*n* = 6), and (3) CNV7d group (*n* = 6), based on the day of sacrifice after laser irradiation.

In the second in vivo experiment, C57BL/6J mice were divided into three groups: (1) mice with intravitreal injection of 1 μl of DMSO (Control group, *n* = 6); (2) mice with intravitreal injection of 1 μl of DMSO one day after laser irradiation (CNV group, *n* = 6); (3) mice with intravitreal injection of 1 μl of Fer-1(30 μM) one day after laser irradiation (Fer-1 group, *n* = 6). After one week, mice were used for subsequent experiments.

In the third in vivo experiment, C57BL/6 J mice were divided into five groups: (1) mice with daily intraperitoneal injection of 200 μl corn oil (Control group, *n* = 6); (2) mice with daily intraperitoneal injection of 200 μl corn oil after laser irradiation (CNV group, *n* = 6); (3) mice with intraperitoneal injection of Vitamin K1 (25 mg/kg) after laser irradiation (VK group, *n* = 6); (4) mice with warfarin sodium through bottled drinking water (0.33 mg/ml water) after laser irradiation (VKA group, *n* = 6); (5) mice with intraperitoneal injection of Vitamin K (20 mg/kg) and warfarin sodium through bottled drinking water (0.33 mg/ml water) after laser irradiation (VK + VKA group, *n* = 6). After one week, mice were used for subsequent experiments.

### Spectral domain optical coherence tomography (SD-OCT) and fundus fluorescein angiography (FFA)

SD-OCT and FFA assessments were conducted at predetermined time points before sacrifice. Prior to examination, mice were deeply anaesthetized, and their pupils were dilated using tropicamide phenylephrine ophthalmic solution (Mydrin; Santen Pharmaceutical, Osaka, Japan). Positioned on the platform, the ocular fundus of the mice was sequentially examined using the SD-OCT system (Heidelberg Engineering, Heidelberg Germany). Subsequently, 50 μl of 10% fluorescein sodium solution (0.1 ml/kg; Alcon Laboratories, Ft. Worth, TX, USA) was administered via intraperitoneal injection. The ocular fundus was then consecutively imaged using a commercial digital fundus camera (Heidelberg Retina Angiograph, Heidelberg, Germany). The fluorescein leakage level was measured by analyzing fluorescence areas and intensity using ImageJ software.

### RPE-choroid flat-mounts

At predetermined time points, mice were euthanized by intraperitoneal injection of an overdose of 2% sodium pentobarbital (200 mg/kg). Eyeballs were extracted for fluorescent-labeled isolectin staining of the CNV lesions. All eyeballs were carefully enucleated, fixed with 4% PFA, and then incubated with a blocking solution comprising 5% Goat serum and 0.3% Triton X-100 in PBS. Then, the eyecups were stained with FITC-conjugated Bandeiraea simplicifolia isolectin B4 (IB4; 1:100; VectorLabs, Newark, CA, USA) in blocking buffer at 4 °C for 8 h, and the nuclei were stained with DAPI (Invitrogen) for 15 min, shielded from light. Following staining, the RPE/choroid/sclera complex was carefully dissected into four incisions and mounted flat on a glass slide with the RPE side facing upward. All flat-mounts images were captured usings a confocal laser scanning microscope (FV3000; Olympus, Tokyo, Japan), and the area of CNV-related fluorescence was quantified using ImageJ software.

### Hematoxylin - eosin (HE) and immunofluorescence (IF) staining

At the predetermined time points, the mice were euthanized, and their eyeballs were carefully extracted and immediately stored in FAS eyeball fixative solution (Servicebio, Wuhan, China). After dehydration in a series of ethanol solutions, the eyeballs were embedded in paraffin. Subsequently, tissue blocks embedded in paraffin were sectioned to a thickness of approximately 5 μm. HE staining was performed on the sections using hematoxylin-eosin. As described previously [6], IF slices were washed with PBS and fixed with goat serum, and then incubated with the antibodies listed in Supplementary Table S1 for 8 h at 4 °C. Images were obtained from at least three regions of each slice using a Nikon optical microscope (Tokyo, Japan). The primary antibody was detected using a fluorescent secondary antibody (Alexa Fluor 488/594; 1:500; Invitrogen) for 1 h at 37 °C. DAPI (Invitrogen) were used to stained the nuclei.

### Transmission electron microscopy (TEM)

Within 2 min of eyeball extraction, a small piece of retina was removed from the posterior pole and fixed at 4 °C with 2.5% glutaraldehyde phosphate (0.1 M, pH 7.4) (Science services, Munich, Germany) for 2 h, followed by fixation with 2% osmium tetroxide for another 2 h. Then samples were dehydrated and embedded in Epon812 (Merck, Darmstadt, Germany). Subsequently, tissues were sectioned to a thickness of 60 nm, stained with lead citrate and uranyl acetate, and examined using transmission electron microscopy. Pictures were obtained using TEM microscopy (FEI, Hillsboro, OR, USA).

### Iron ion detection

RPE cells were collected following the manufacturer’s instructions of the Iron Assay Kit. The absorbance at a wavelength of 593 nm was measured using a microplate reader (Multiskan skyhigh, Thermo Fisher Scientific, Waltham, MA, USA). The concentration of ferrous iron was calculated based on a standard curve.

### GSH/GSSG and MDA measurement

After collecting the RPE cells, they were lysed by ultrasonication at 0 °C for 20 min, followed by centrifugation at 4 °C at 15,000 × *g* for 10 min. The cleared supernatant was used for subsequent detection. The GSH content and GSH/oxidized glutathione (GSSG) ratio were measured using a GSH/GSSG Quantification Kit according to the manufacturer’s instructions. The Malondialdehyde (MDA) content was assessed using an MDA Assay Kit following the manufacturer’s instructions. Total protein concentration was determined using a BCA Protein Assay Kit (Thermo Fisher Scientific).

### Western blot analysis

Total protein was extracted using RIPA buffer (Invitrogen) containing 1% proteinase inhibitors (New Cell & Molecular Biotech, Suzhou, China) on ice, and the lysates were sonicated. Protein concentrations were determined using a BCA Protein Assay Kit.

The lysate proteins were isolated by SDS-PAGE gels (Bio-Rad, Hercules, CA, USA) and transferred onto PVDF membranes (Merck). After blocking with 5% BSA for 1 h, the membranes were incubated at 4 °C with different primary antibodies listed in Supplementary Table S2. Subsequently, the membranes were washed three times with TBST (pH 7.4, 10 mM Tris–HCl, 150 mM NaCl, and 0.1% Tween 20) and then incubated with horseradish peroxidase conjugated secondary anti-rabbit/mouse antibodies (1:5,000; Cell Signaling Technology, Danvers, MA, USA). Protein signals was viewed using an ECL Plus Western Blot Detection Kit (Tanon, Shanghai, China).

### Cell culture and drug treatment

The human RPE cell line, ARPE-19 cells, obtained from the Cell Bank of the Chinese Academy of Sciences (Shanghai, China), and primary HUVEC provided by ScienCell Research Laboratories (Carlsbad, CA, USA) at passages 3–8 were used. The culture medium comprised DMEM, 10% FBS, and 1% penicillin/streptomycin, with medium changes every 2 days prior to conducting experiments.

Erastin (dissolved in DMSO, 1, 3, 5, 10, 20 μM) was used to induce ferroptosis cell death. Fer-1 (dissolved in DMSO, 10 μM) and Vitamin K1 (dissolved in ethyl alcohol, 0.1, 1, 3, 5, 10, 20, 25, 30, 50, 100 μM) were added to the culture medium 1 h before treating with ferroptosis activator. Warfarin (dissolved in DMSO, 1, 5, 10 μM), iFSP1 (dissolved in DMSO, 10 μM), and ISRIB (dissolved in DMSO, 10 μM) were added during cell seeding.

In the HUVEC conditioned culture experiments, the conditioned medium consisted of ARPE-19 cell supernatant. Specifically, ARPE-19 cells were cultured on the upper chambers of 0.4 μm-pore 6-well Costar Transwells for over 42 days to confluence and mature [37], following stimulation experiments as indicated for another 24 h. Then the culture medium was replaced with fresh medium and the supernatant was collected from the basolateral Transwell compartments after 24 h. Then, the scratch migration and tube formation assays of HUVEC were performed following incubation with the conditioned medium for 24 h. The concentration of FBS in the ARPE-19 culture medium for the scratch migration assay was 2%.

### CCK-8 assays

The CCK-8 kit was used to evaluate the viability of ARPE-19 cells. Cells (2 × 10^4^ cells/well) were seeded in 48-well plates and treated as indicated. Subsequently, the cells were digested using pancreatic enzymes, centrifuged, and suspended in equal volumes, and seeded in equal volumes into 96-well plates. Then, 10 µL CCK-8 reagent was added to each well and incubated at 37 °C for 4 h. The absorbance at 450 nm was measured using a microplate reader (ELX800, BioTek, Suwanee, GA, USA). Five independent experiments were performed.

### Live/dead staining

A Live/dead Kit was used to stain the ARPE-19 cells (4 × 10^4^ cells per well) cultured in a 24-well plate for the determination of viability, as directed by manufacturer’s instructions. At the determined time, the cells were incubated with Calcein AM and PI reagents for 15 min, and pictures were captured using a fluorescence microscope (Nikon).

### ROS assays

The ROS level was evaluated using a highly Sensitive DCFH-DA-ROS assay kit following the manufacturer’s instructions. In brief, cells were washed twice with HBSS and then incubated with the highly sensitive DCFH-DA dye working solution for 30 min. Fluorescence signals were obtained using confocal laser scanning microscopy (FV3000, Olympus).

### Lipid peroxidation assessment

ARPE-19 cells were treated as indicated and incubated for 1 h, protected from light, with a 50 μM Lipid Peroxidation Probe BDP 581/591 C11. After washing twice with PBS to remove excess probes, a small amount of serum-free medium was added to cover the cells in the dish. Fluorescence signals were obtained using confocal laser scanning microscopy (FV3000, Olympus).

### Mitochondrial membrane potential detection

Mitochondrial membrane potential was evaluated using a JC-1 MitoMP Detection Kit according to the manufacturer’s instructions. After 4 h of erastin treatment, JC-1 solution was added to the culture medium to a final concentration of 4 μM and then incubated at 37 °C for 30 min. Following washing twice with HBSS, the cells were incubated in Imaging Buffer Solution, and fluorescence signals were obtained using confocal laser scanning microscopy (FV3000, Olympus).

### RNA extraction and quantitative polymerase chain reaction (qPCR)

RNA extraction and quantitative PCR were performed as previously described [39]. Total RNA from ARPE-19 cells (5 × 10^5^) in 6-well plates was extracted using TRIzol reagent (Takara, Kyoto, Japan). Reverse transcription of RNA into cDNA was carried out using PrimeScript RT master mix (Takara). To determine mRNA expression levels, real-time qPCR was performed using a Real-Time PCR Detection System (Applied Biosystems, Foster, CA, USA) according to the manufacturer’s instructions. The relative gene expression was analyzed by the 2 − ΔΔCt method. The relative mRNA expression is expressed as the fold change relative to GAPDH expression to ensure accurate gene quantification. Primer sequences were listed in Supplementary Table S3.

### ELISA quantification

ARPE-19 cells were cultured on the upper chambers of 0.4 μm-pore 6-well Costar Transwells for over 42 days to confluence and mature, and then treated as indicated for 24 h. The culture medium was refreshed 24 h before the supernatant was collected from the basolateral Transwell compartments. The concentration of VEGFA was measured by an ELISA quantification kit (ABclonal, Wuhan, China).

### Scratch migration assay

When HUVEC cells reached 100% confluence in 6-well plates, a wound injury was created by scratching the cell monolayer using disposable 200 μl pipette tips. Then, PBS was used to wash the cell debris away, and the cells were incubated in the corresponding ARPE-19 conditioned medium. A Nikon digital camera screened all the pictures of wound healing assays. Images of wound healing were taken per 12 h, and we chose the images of 0 h, 12 h, 24 h to assess the lateral migration ability of cells. Experiments were repeated three times, and the final scratch fusion area was calculated using ImageJ software.

### Tube formation assay

The tube formation assay was conducted following established protocols [40]. Matrigel (Corning, USA) was pre-coated onto 96-well plates, and resuspended HUVEC cells were seeded into each well with conditioned medium composed of ARPE-19 cell supernatant treated as indicated. After incubated for 8 h, five random fields from each well were imaged using a microscope (Nikon). The experiments were repeated three times, and tube formation ability was quantified by measuring the number of nodes, tubes, and total segment length using ImageJ software.

### RNA-sequencing and analysis

Total RNA of ARPE-19 cells was extracted and reverse-transcribed into cDNA using TRIzol reagent and PrimeScript RT master mix, as previously described [6]. The ligated products were amplified with PCR following a series of pretreatments, with an average insert size of 300 ± 50 bp for the final cDNA library. The 2 × 150 bp paired-end sequencing (PE150) was performed on an Illumina Novaseq™ 6000 (LC-Bio Technology Co., Ltd., Hangzhou, China). The RNA sequencing data has been deposited in the National Center for Biotechnology Information’s Gene Expression Omnibus (GEO) database.

### siRNA transfection

ARPE-19 cells were seeded in 6-well plates for 24 h and transfected with siRNA (Gene Pharma, Shanghai, China) following the manufacturer’s instructions. The siRNA sequences used in this study were siATF4 (Qiagen, SI03019345), siVKORC1L1(Qiagen, SI04138407), siVKORC1(Qiagen, SI00760074), siFSP1(Qiagen, SI03082541), and siNC (Qiagen, SI03650318). The cells were transfected with 50 nM siRNAs using Lipofectamine® 3000 kit (Thermo Fisher Scientific) for 8 h at 37 °C in a 5% CO2 incubator. Following transfection, the culture medium was refreshed with complete medium, and the cells were reseeded in the complete culture medium for at least 8 h for subsequent experiments.

### Statistical analysis

Each experiment was repeated at least three times unless otherwise stated. Statistics were display as the mean ± standard deviation (SD). Statistical analysis was performed using Student’s *t* test for comparisons between two groups or one-way analysis of variance (ANOVA) with post hoc Bonferroni correction for comparisons among >2 groups. A value of *P* < 0.05 was considered statistically significant.

## Supplementary information

Figure legend Supplementary Figs

Fig.S1

Fig.S2

Fig.S3

Table S1

Table S2

Table S3

Reproducibility checklist

## Data Availability

The raw RNA-seq data reported in this study have been deposited in the GEO database with the accession number GSE267133, and are publicly accessible at. The original western blots reported in this study are shown in Supplementary Fig. S3.
