# Supplementary material for: The role of vitamin K and its antagonist in the process of ferroptosis-damaged RPE-mediated CNV
Source: Cell Death Dis. 2025 Mar 20;16(1):190. doi: 10.1038/s41419-025-07497-0 (PMC11923134; doi:10.1038/s41419-025-07497-0)
Supplement: Supplementary file 1 — Figure legend Supplementary Figs [file 41419_2025_7497_MOESM1_ESM.docx]

Supplementary Figs. S1. (A) Protein levels of ATF4 in ARPE-19 treated as indicated were examined by western blot. (B) Protein levels of VKORC1 and VKORC1L1 in ARPE-19 treated as indicated were examined by western blot. (C) Protein levels of FSP1 in ARPE-19 treated as indicated were examined by western blot. The data represent the averages of three independent experiments.

Supplementary Figs. S2. Schematic diagram of inhibiting neovascularization in ferroptosis-damaged RPE cells through eIF2α-ATF4-VEGFA.

Supplementary Figs. S3. The original western blots reported in this study.
