# Supplementary figures and images for: The role of vitamin K and its antagonist in the process of ferroptosis-damaged RPE-mediated CNV
Source: Cell Death Dis. 2025 Mar 20;16(1):190. doi: 10.1038/s41419-025-07497-0 (PMC11923134; doi:10.1038/s41419-025-07497-0)

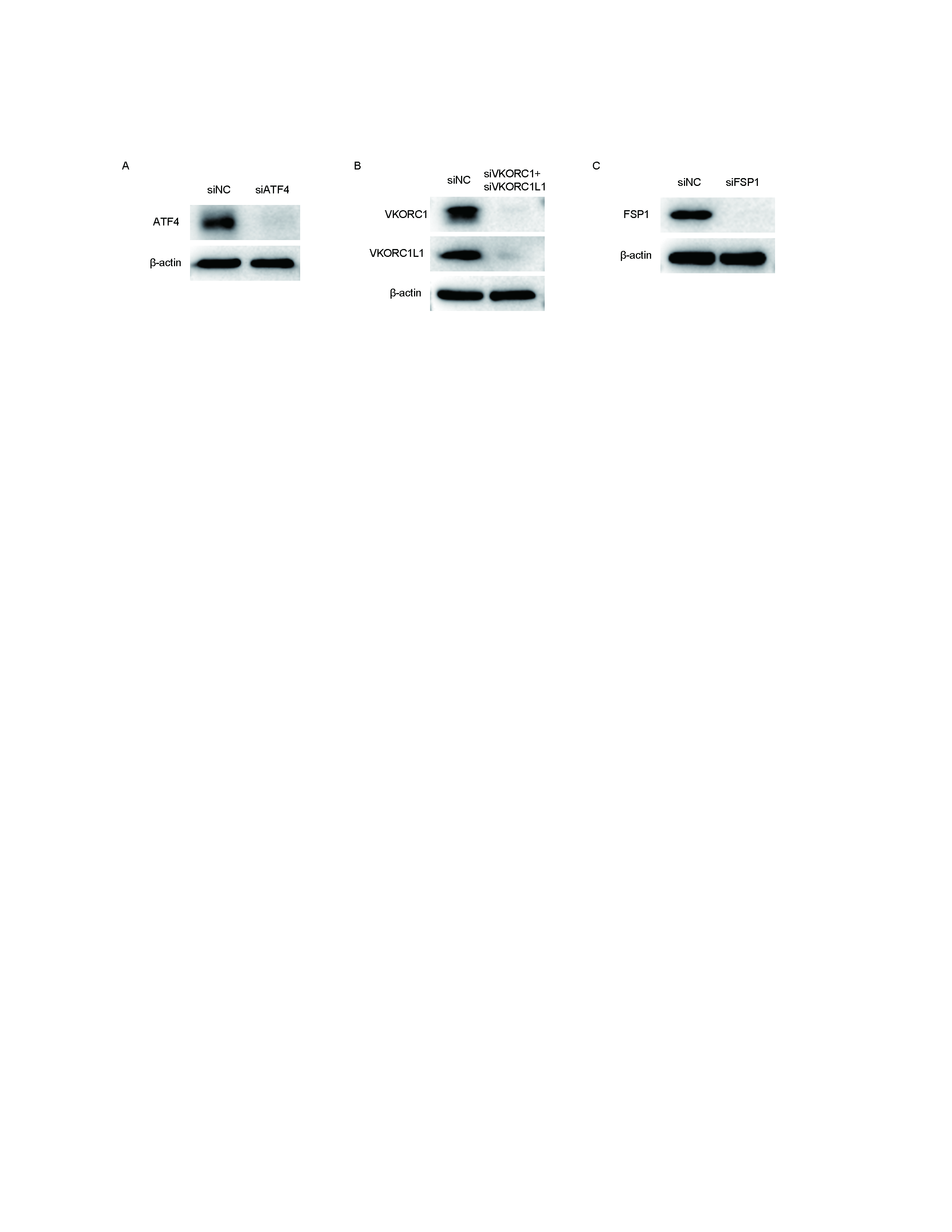

Supplement: Supplementary file 2 — Fig.S1 [file 41419_2025_7497_MOESM2_ESM.tif]

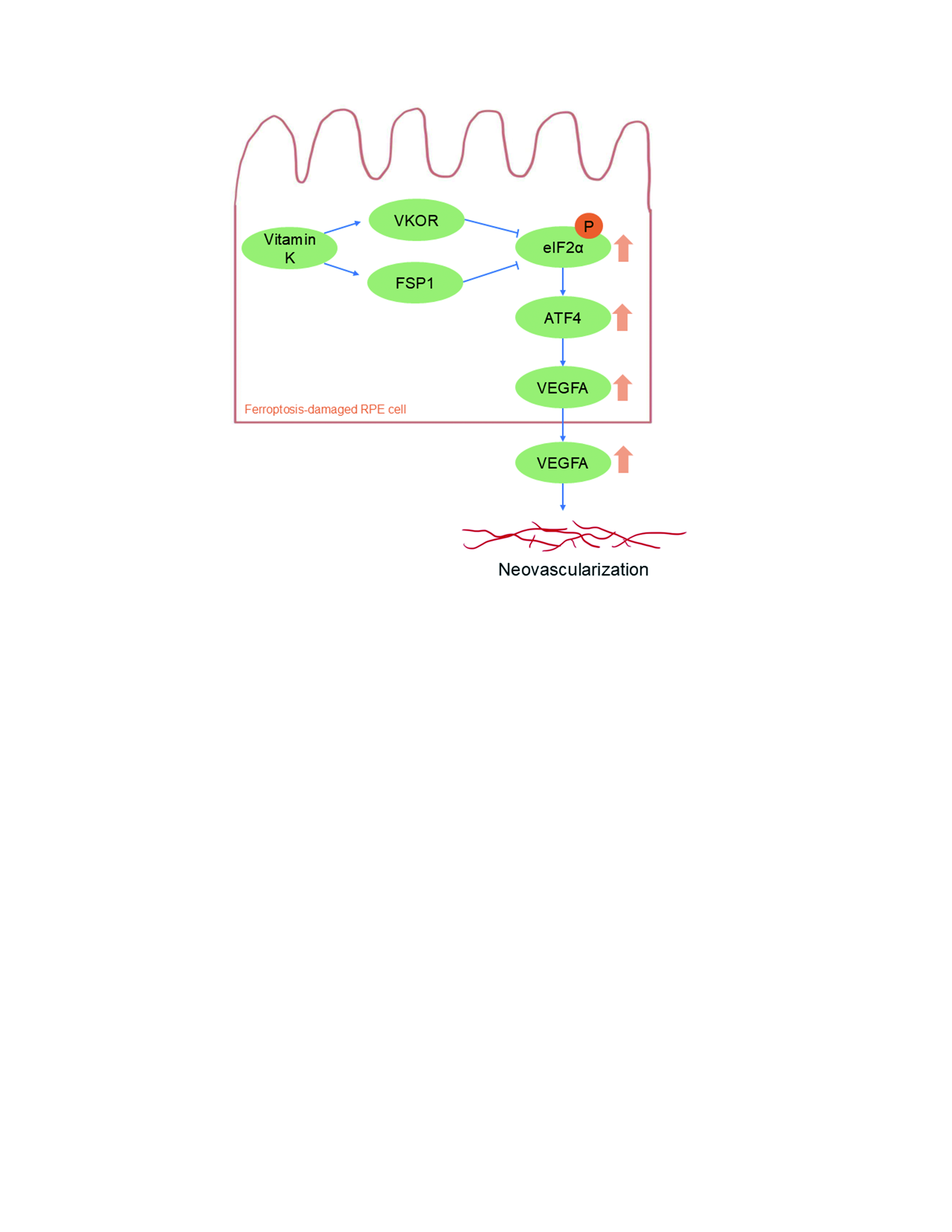

Supplement: Supplementary file 3 — Fig.S2 [file 41419_2025_7497_MOESM3_ESM.tif]

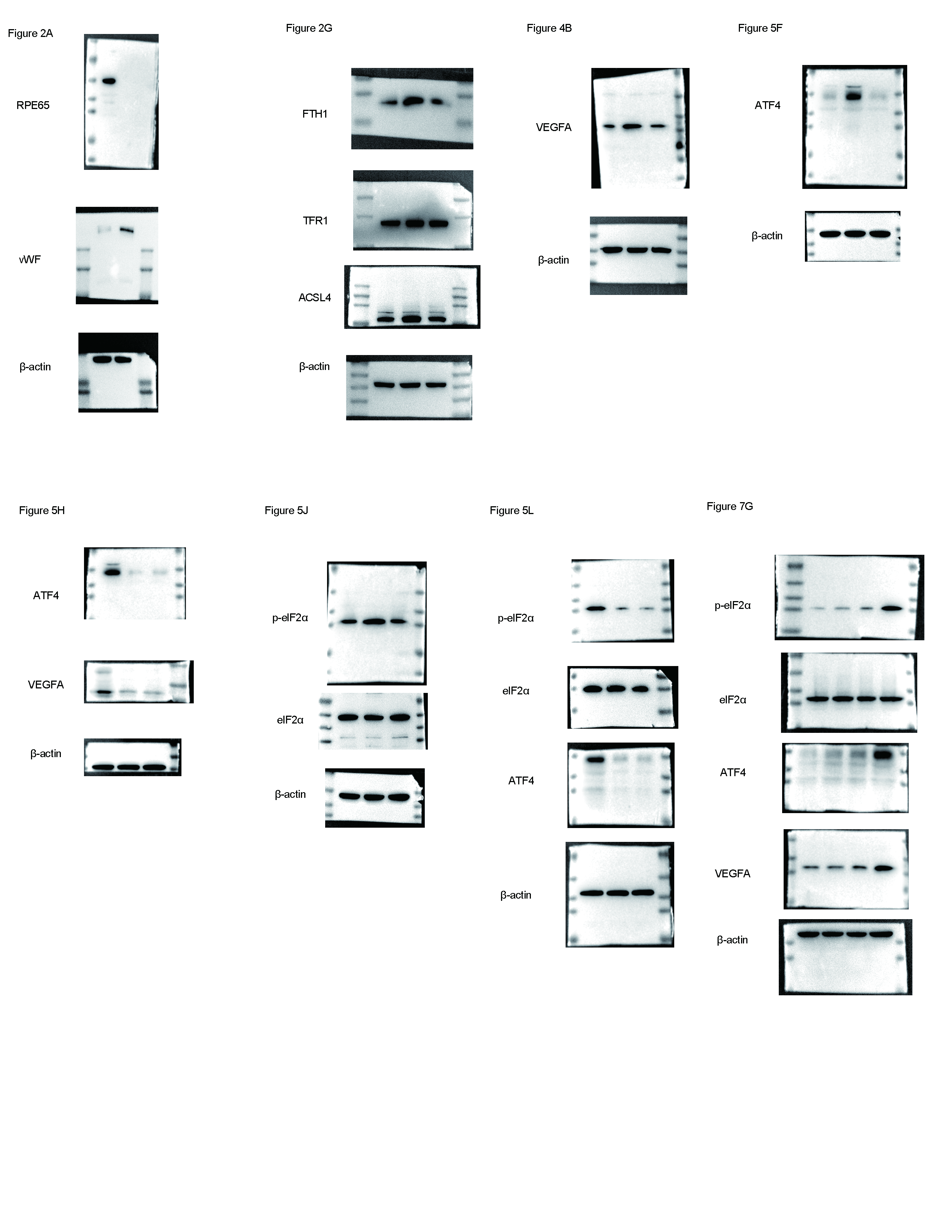

Supplement: Supplementary file 4 — Fig.S3 [file 41419_2025_7497_MOESM4_ESM.tif]
